# Development and evaluation of a hand held computer based on-call pack for health protection out of hours duty: A pilot study

**DOI:** 10.1186/1471-2458-5-35

**Published:** 2005-04-11

**Authors:** Ibrahim Abubakar, Christopher J Williams, Marian McEvoy

**Affiliations:** 1 Health Protection Agency (East of England) Regional Epidemiology Unit, Institute of Public Health, Cambridge, CB2 2SR, UK; 2 School of Medicine, Health Policy and Practice, University of East Anglia, Norwich, NR4 7TJ, UK; 3 Health Protection Agency, East and North Hertfordshire Health Protection Unit, Charter House, Welwyn Garden City, UK

## Abstract

**Background:**

The on call service for health protection in most parts of the UK is provided by general public health consultants, registrars and nurses as the first tier of response backed up by medical consultants in health protection. The first tier responder usually carries a large bag of papers containing both local and national guidance on the management of common cases/incidents. An electronic on call pack may provide a suitable practical alternative to large paper based systems and help professionals deliver out of hours health protection advice and response to incidents.

**Methods:**

We developed and piloted an electronic on call pack in Hertfordshire for use at the health protection unit level containing key local and national guidelines, contact information and useful references. The on-call pack was initially piloted using a laptop and more recently using a personal digital assistant (PDA). The use of the on-call pack was evaluated.

**Results:**

Key advantages of the electronic system include reduced size, faster access to information that is clearly indexed and the relative ease of updating information. As part of the pilot, the electronic on call pack was presented to a local and regional training meeting with good response from participants using qualitative and quantitative methods.

**Conclusion:**

It is anticipated that with suitable evaluation this system can be adapted and utilised by other health protection practitioners. This system provides a fast, reliable and easily maintained source of information for the public health on-call team.

## Background

Personal Digital Assistants (PDAs) or handheld computers are now commonly used devices in clinical medicine and other professions where immediate access to information is required away from the comforts of a traditional office and desk top computer [1].

Several specialities of clinical medicine are using PDAs for the delivery of information at the point of care including anaesthesia [2], surgery [3], paediatrics [4], general practice[5] and obstetrics [6]. PDAs have also been used to collect patient information and improve clinical records, for administrative functions such as electronic prescribing [7], coding and tracking [8], in research projects. [9-11] and in medical education including the monitoring of training [12]. This paper describes the piloting of a PDA in the on call health protection service.

Health protection is a part of public health and is defined as those public health activities intended to protect individuals, groups, and populations from infectious diseases, environmental hazards such as chemical contamination, and from radiation [13]. The on call service for health protection varies in different parts of England. The UK Faculty of Public Health in collaboration with other UK professional and governmental bodies including the Health Protection Agency (HPA), Department of Health, Association of Directors of Public Health and Public Health Medicine Environmental Group has recently published recommendations for the organisation and delivery of out of hours service to protect the public's health [14]. In Hertfordshire, as in many parts of the UK, the on call service is provided by public health registrars and communicable disease control nurses as the first tier of response backed by public health/health protection consultants as the second tier. The first on call was previously required to carry a large bag of papers containing both local and national guidance on the management of common cases/incidents. Managing incidents may require the responder to visit incident sites located away from a normal office setting where access to information is easy.

The reasons for developing an electronic on call pack in Hertfordshire were to improve portability by reducing the size and weight of the on call pack and to enable easier updating. The training group of the Local and Regional Services Division of the HPA has developed a number of initiatives to facilitate and improve the quality of training provided for staff of the Agency. This on call device may contribute to the effort of the Agency in delivering this function by providing an additional option for those on call to be more effective.

The aim of this study was to develop and pilot the use of a portable electronic on call pack for use by the public health on-call team in Hertfordshire to deliver the health protection function. The advantages, disadvantages and feasibility of using this system were assessed.

## Methods

### Developing the on-call pack

The East and North Hertfordshire paper based on call pack was converted into electronic text files using Microsoft Frontpage^© ^in January 2003.

### Software and hardware

The on call pack was loaded from the desktop computer where it was initially developed unto a pocket PC based IPAQ^© ^PDA by synchronising. All guidelines were in portable document format (.pdf) and could be view ed with an Adobe Reader^© ^for pocket PC. Local information files were all Microsoft Word^© ^documents and could therefore be easily updated. The choice of Pocket PC operating system was based on ease of adaptation of documents already prepared for use with Microsoft software. The process of adaptation entails automatically converting files from a PC based version, for instance, Microsoft Word documents were converted to a pocket PC equivalent and where appropriate the formatting and content were changed. A Palm based on-call pack is currently under development.

### Structure and content

**Figure 1:**
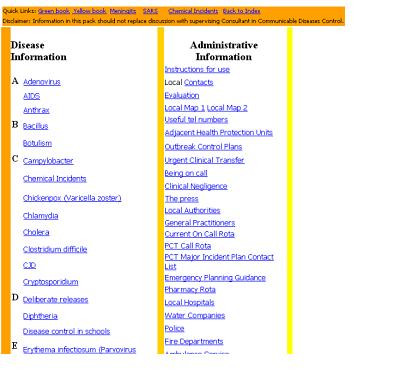
Structure of the on call pack as viewed on a PDA

**Figure 2:**
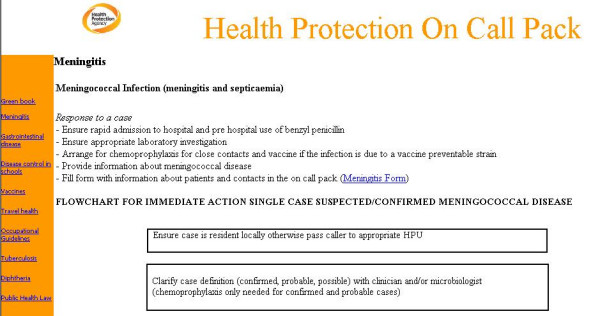
Immediate on call action for meningococcal disease

**Figure 3:**
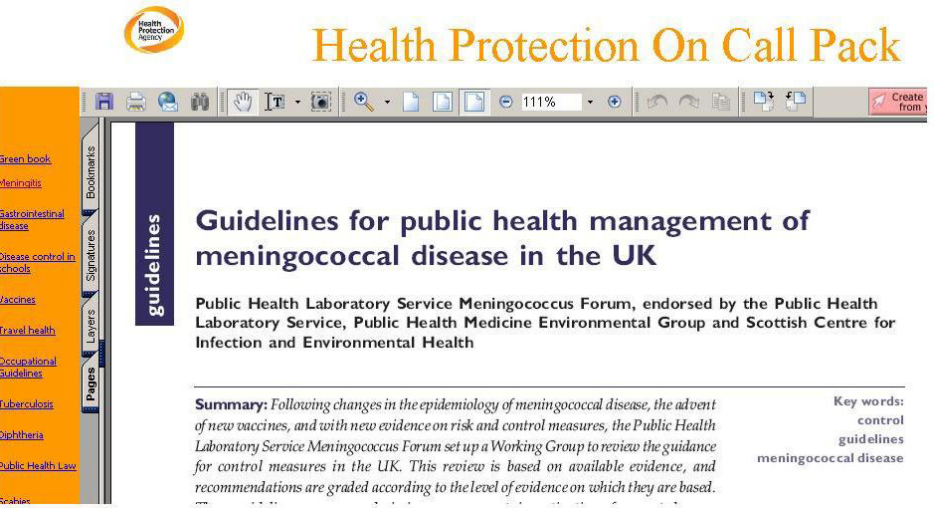
UK National guidelines for meningococcal disease

The on-call pack consists of an index page, which can either be on a laptop or a PDA. The main index is divided into a disease information column and an administrative information column (figure 1). Each disease page was divided in to three sections, 1. on-call action summarising key steps to be taken when responding to a call (figure 2), 2. additional information for managing cases/incidents and 3. links to further national or local guidelines for the management of the specific condition that can be accessed without connecting to the internet (figure 3). The administrative information section consists of contact details, on call rotas for different organisations and how to deal with the media. A user guide was included linked to the administrative information column. The main differences between the PDA and laptop versions of the on call pack include the smaller screen on the PDA and therefore bigger font required and different versions of the software used.

### Sources of information

The information in the on-call pack is based primarily on data already in the paper version of the pack in three health protection units in England (Hertfordshire, Cambridgeshire and Bedfordshire). We used national guidelines for the management of communicable disease cases and outbreaks, chemical and radiological incidents and emergency planning guidance as published on the HPA [15], National Electronic Library for Infections [16] and UK Department of Health's [17] websites. We obtained further information from textbooks commonly used for the public health management of communicable diseases including Communicable Disease Control Handbook [18] and Chin's Communicable Disease Control Manual [19]. Although the authors assessed each source prior to inclusion no formal evaluation of the sources was conducted.

### Updating information in the on-call pack

The administrative information documents such as the on-call rota can be updated by either replacing the word document or cutting and pasting content into the document. The content of disease-based pages can be updated with any text editor such as Microsoft Notepad^© ^or with web editing software such as Frontpage^© ^or Dreamweaver^©^. Arrangements for updating the content of the on-call pack have not been agreed and are currently undertaken on an ad-hoc basis. Ideally a formal process for regular updating should be implemented because the current ad-hoc system is not satisfactory.

### Pilot

The use of the on-call pack was evaluated in a pilot study over the first year of its development initially as a tool on a laptop computer and subsequently on a PDA. The components of the evaluation includes a qualitative and a quantitative aspect.

### Qualitative

#### District

The on-call pack was presented at a training meeting for trainees and consultants on the district on-call rota held in February 2003. The presentation was used to introduce the on-call pack on a laptop computer and to obtain feedback regarding its potential usefulness and whether individuals on the rota were willing to use the pack as an alternative to the current system. Attendees asked questions and gave comments at the end of the presentation which were used to improve the next version of the pack.

#### Regional

The on call pack was presented at a regional training event held in April 2004 for general public health consultants and directors of public health. The content of the pack was explained and attendees were allowed to review the PDA. A questionnaire was given asking for their views on how useful they thought it would be. The evaluation questionnaire was based on a Likert scale of good, fair, neutral, weak and poor and free text comments.

### Quantitative

This aspect of the evaluation was carried out over a period of five weeks, each person on duty was given the on-call pack at the beginning of the week. Three PDAs were used so individuals had to return the device at the end of the on-call period. Each person on-call was given an evaluation questionnaire to be completed at the end of the week. Questions were asked to comment on the frequency of use, type of incidents, ease of access, quality of information and portability. Responses were collated and analysed in a spreadsheet. Quantitative measures were summarised using an average score and range, while free text comments were summarised in a table.

## Results

### Qualitative

#### District

Several comments were received from the district training day. The main suggestions were; firstly, there is a need for a back up facility as with any electronic device as failures can happen. Secondly, a search function to identify key documents without the need to scroll through several pages would be helpful. Thirdly, there were concerns about confidentiality issues if a PDA or laptop was to be used for recording patient identifiable information and fourthly, participants were concerned about how the on-call pack would be updated.

#### Regional

Twenty individuals attended the regional training meeting and 12 completed evaluation forms. Eleven of the 12 respondents scored the on-call pack "good", while one gave it a "fair". Comments included a suggestion that training on using the on call pack should be provided for anyone going on the rota and that contents should be available on a web page to synchronise with individual PDA's. Potential difficulties highlighted include problems with batteries running down, the need for a paper backup and difficulty in reading some documents with very small font.

### Quantitative

Five individuals trialled the PDA based on-call pack over a five week period, four of whom completed and returned evaluation forms (participants A, B, C, D). The mean number of calls answered was 1.5 (range 0–3 calls). Incidents tackled during the week varied and included both infections and chemical incidents. Table one summarises the response of the participants.

Assessment was based on a Likert scale of one to five where one was poor, three was same as paper version and five was very good. All three respondents gave a score of good for ease of use and very good for portability. The quality of content was rated four out of five by all three respondents.

The coordinator of the evaluation had difficulties with handover of the limited number of PDA's and with synchronising PDAs to the desktop computer.

## Discussion

We have developed and piloted the use of a handheld computer based on-call pack for health protection out of hours duty. As far as we are aware, this is the first use and evaluation of a hand held device for public health on call duties. The idea of using an electronic portable pack was considered good and acceptable by most individuals on the rota and the information was found to be easy to access and use.

PDAs have been found to be useful in delivering information at the point of use in several specialities of clinical medicine[1]. The findings of this study indicate that many of the benefits of using PDAs in clinical medicine could be applicable to public health practice. Availability of information is especially important because of the increasing volume of knowledge and the expectations that guidelines will be followed. Other aspects of the basic functions of a PDA can provide added benefit to the reference function intended in our application. These functions include the address book, scheduler, to do list and memo [1]. There is also a voice recorder that can be used to record notes for subsequent typing.

There are several advantages of using a PDA identified from this and other studies. Portability was found to be one of the main positive attributes of the on-call pack as users found the device more convenient to carry compared to the paper based version. This was the biggest strength of the device given the amount of information required for dealing with incidents. An advantage cited by users is the ease of access to information. This has been reported in other studies[20,21] It would be useful to compare the outcome of incidents and assess whether using a handheld device improves outcome. We have not conducted an outcome evaluation due to the long-term nature of results of public health measures and resource limitation. The results of the evaluation should be interpreted cautiously due to the small sample size.

Ideally each person on call should be provided with an individual PDA but cost considerations are important. Each PDA used cost one hundred and ninety nine pounds, but simpler versions are available for less. There was no additional cost for software. The time of a specialist registrar was required to update and maintain the information. The costs of the paper based on-call pack included the cost of a bag, papers, printing and time of the person updating and printing the information.

Disadvantages of using a PDA based on call pack include occasional problems with reading information due to small size of the device. The quality of the information in the pack was rated good rather than very good which means it can benefit from further improvement. The content of the pack would benefit from an editorial group. The on-call action for each disease can be replaced with agreed standard operating procedures. Other areas that need to be addressed before such a scheme is used more widely include the provision of a back up facility and the availability of an efficient system to update the pack. The previous paper based on call pack contained a large volume of guidelines which made it difficult to comprehensively catalogue and update. The initial update during conversion of the pack to an electronic format made explicit the need for continual updating. The most feasible way to update a regional or national on-call pack would be through a web based password protected server. The administrative content can be updated locally. The provision of training and good administrative and information technology support will also be essential as reported by other authors [20,21]. Any support system developed should be linked to local services.

Comparing the use of PDAs and laptop computers in our study suggests that PDAs were more likely to be used because of portability. Although smaller than the paper based on call pack, most laptop computers are too large to carry around. PDAs are significantly cheaper than laptops, while laptop computers do not require synchronisation because data can be updated directly.

The use of PDAs is widespread among junior doctors especially in North America. Evidence suggests there is greater use among younger physicians [1,22]. This has implications for use in health protection. A significant number of future public health doctors will start public health training with previous experience of using PDAs as these devices are increasing used at all levels [23] and therefore, facilitate access. Conversely, older doctors may find it difficult to adapt to the change. Reasons given by older physicians for not using PDAs include the small size of the touch screen typing device which is difficult to use [24] and lack of familiarity with computer systems. In a recent qualitative study, McAlearney et al found the main barriers to the use of handheld computers to fall in two broad categories, physical (older age, poor sight and finger size) and perceptual (low comfort with technology and devices, preference for paper or desktop computers) constraints [20]. The difficulty with visualising text on PDAs can be aided by using a larger font while that with data entry can be tackled by either using a portable keyboard or with the aid of a voice recording device.

Further functions can be added to the device to enhance it use including a search function, internet access and data entry facilitating surveillance and potentially contributing to electronic medical records in the future. The most significant constraint to data entry will be security. There are software available to encrypt data on a PDA [23].

The pack was specifically developed for public health on call staff and general public heath consultants. Due to the potential for memory expansion a significantly larger volume of reference texts and books could be added making the device useful for all cadres of health protection staff. Increasing number of reference texts are becoming available via PDAs. For example, textbooks such as the Oxford Handbooks and the British National Formulary are available on a hand held device. Several journals and Pubmed have also developed PDA compatible access.

We conclude that a PDA could be a useful addition to tools available for efficiently delivering information at the point of use for out of hours health protection on call duty.

## Competing interests

The author(s) declare that they have no competing interests.

## Authors' contributions

IA conceived the idea and developed the on call pack and drafted the manuscript, CJW carried out the evaluation. All the authors participated in writing the paper.

**Table 1:** Summary of responses from participants

Participant	A	B	C	D
Number of calls/week	0	1	2	3
Frequency of use	0	2*	2	2
Ability to find specific information	N/A	Yes	Yes	Yes
Information used	N/A	Measles advice	Immunoglobulin & vaccination advice, E. coli, Hep B, measles, chicken pox, useful numbers	chemical incidents and meningitis
Comments	"Contacts was missing on this PDA" "PDF files difficult to read" "Travel immunisation advice useful"	"Needs regular updating as no paper backup"	"IPAQ is the way forward rather than carrying a laptop I have no computer at home PDA makes it easier to have a life and go out" "Would like some training on all functions"	"Extremely good idea. Information easy to find. Some files not accessible, contact info not available Some tables in guidelines (measles) too small to read and couldn't magnify" "Using PDA far preferable to laptop"

* including one use for familiarisation

## Pre-publication history

The pre-publication history for this paper can be accessed here:


